# Effects of transcranial direct current stimulation of left and right inferior frontal gyrus on creative divergent thinking are moderated by changes in inhibition control

**DOI:** 10.1007/s00429-020-02081-y

**Published:** 2020-06-17

**Authors:** Radwa Khalil, Ahmed A. Karim, Angela Kondinska, Ben Godde

**Affiliations:** 1 Department of Psychology and Methods, Jacobs University Bremen, Bremen, Germany; 2 Department of Health Psychology and Neurorehabilitation, SRH Mobile University, Riedlingen, Germany; 3 Department of Psychiatry and Psychotherapy, University of Tübingen, Tübingen, Germany

**Keywords:** Creativity, Divergent thinking (DT), AUT (alternative uses task), Fluency, Originality, Flexibility, tDCS, IFG

## Abstract

Divergent thinking (DT) as one component of creativity is the ability to search for multiple solutions to a single problem and is reliably tested with the Alternative Uses Task (AUT). DT depends on activity in the inferior frontal gyrus (IFG), a prefrontal region that has also been associated with inhibitory control (IC). Experimentally manipulating IC through transcranial direct current stimulation (tDCS) led to alterations in DT. Here, we aimed at further examining such potential mediating effects of IC on DT (measured as flexibility, fluency, and originality in the AUT) by modulating IC tDCS. Participants received either cathodal tDCS (c-tDCS) of the left IFG coupled with anodal tDCS (a-tDCS) of the right IFG (L−R + ; *N* = 19), or the opposite treatment (L + R−; *N* = 21). We hypothesized that L + R− stimulation would enhance IC assessed with the Go NoGo task (GNGT), and that facilitated IC would result in lower creativity scores. The reversed stimulation arrangement (i.e., L− R +) should result in higher creativity scores. We found that tDCS only affected the originality component of the AUT but not flexibility or fluency. We also found no effects on IC, and thus, the mediation effect of IC could not be confirmed. However, we observed a moderation effect: inhibition of left and facilitation of right IFG (L−R +) resulted in enhanced flexibility and originality scores, only when IC performance was also improved. We conclude that inducing a right-to-left gradient in IFG activity by tDCS is efficient in enhancing DT, but only under conditions where tDCS is sufficient to alter IC performance as well.

**Electronic supplementary material:**

The online version of this article (10.1007/s00429-020-02081-y) contains supplementary material, which is available to authorized users.

## Introduction

Creativity and innovative thinking in the arts, science, stage performance, the commercial enterprise, and business innovation is a multidimensional construct (Kaufman 2007) and based on diverse psychological and cognitive processes (Csikszentmihalyi 1999; Kaufman and Beghetto 2009; Gaut 2010; Sawyer 2011; Perlovsky and Levine 2012; Khalil et al. 2019). Explicitly, creative cognition is at the root of extraordinary performance in arts and sciences (Baas et al. 2015). There are certain types of creative processes, such as divergent thinking (DT) and convergent thinking (CT), that are selectively affected by inhibitory control (IC) (Radel et al. 2015; Cassotti et al. 2016; Khalil et al. 2019).

IC is a central component of executive function (EF) and allows the suppression of automatic, prepotent, or inappropriate actions and ideas (Aron et al. 2014). This inhibition of automatic and prepotent thoughts, actions, and responses is crucial for creativity (Benedek et al. 2014; Radel et al. 2015). An association between IC and creative performance has been established (for review, cf. Khalil et al. (2019)). For example, Radel et al. (2015) exposed their participants to an Eriksen Flanker (Eriksen and Eriksen 1974) or Simon (Simon 1990) task before performing creativity tests. In these cognitive tasks, perception of (Eriksen Flanker) or response to (Simon) stimuli distracting from the target stimuli need to be inhibited. Exhausting the participants' inhibitory control resources by these tasks led to enhanced fluency (i.e., number of ideas) and originality (i.e., generation of uncommon ideas) in the Alternative Uses Task (AUT; Carroll and Guilford (1968)) but not the Remote Associate Task (RAT; Mednick (1962)). AUT and RAT are well-established measures for DT and CT, respectively. Thus, a lack of resources for inhibition might lead to facilitation of the novelty (i.e., originality) of thoughts (i.e., ideas). Accordingly, one could hypothesize that particular idea generation processes profit from a depletion of resources for inhibition.

The prefrontal cortex (PFC) is known to be part of a deliberate inhibition control network and considered to be a central node for problem solving and idea generation from adolescence to adulthood (Cassotti et al. 2016). Goghari and MacDonald (2009) postulated that a shared prefrontal network (including left and right IFG) mainly serves for processes of response selection and inhibition. However, inhibition is a diverse cognitive dimension, and different sub-dimensions have been attributed to various, distinct frontal cortical sites and hemispheres. Also, the degree of the recruitment of such networks dramatically depends on specific task demands (Aron et al. 2014).

For instance, the left IFG is commonly involved when controlled responses are required, while the right IFG is activated more when task demands are more robust for response inhibition (Goghari and MacDonald 2009). Also, Swick et al. (2008) indicated a role of the left IFG for the successful implementation of IC over motor responses. Whereas the right IFG might also involve response inhibition but is triggered through automatic, bottom-up processing. For example, engagement of right IFG was reported for stop-signal tasks (SST) and interpreted as reprogramming action plans (Lenartowicz et al. 2011). Moreover, Aron et al. (2014) affirmed inhibition as a central component of executive control that depends upon the right IFG and associated networks.

Using transcranial direct current stimulation (tDCS), Cunillera et al. (2014) revealed the involvement of the right IFG in two kinds of inhibition processes (i.e., reactive and proactive inhibition). tDCS is a non-invasive tool that modulates brain function through hyper- or hypopolarization of neurons (Stagg and Nitsche 2011; Medeiros et al. 2012). Stramaccia and coauthors reported evidence about the role of the right IFG in inhibition accuracy in SST (Stramaccia et al. 2015) and, in interference control during memory retrieval processes (Stramaccia et al. 2017).

Even training and developmental studies have hinted to a pivotal role of the IFG in IC. Practicing inhibition tasks reduced the neural activity within the prefrontal inhibitory networks to inhibition trials reaffirming the role of the prefrontal cortex, especially the IFG, in IC (Manuel et al. 2013; Berkman et al. 2014; Chavan et al. 2015). Also, Hartmann et al. (2016) reported enhanced activity of the right frontal cortex, including right IFG, in association with top-down IC after Go NoGo task (GNGT) training. GNGT is a mutual task used to assess IC in humans and animals where participants have to respond quickly to frequently occurring ‘Go’ stimuli and to inhibit responses to infrequent ‘NoGo’ stimuli (Tamm et al. 2002; Swick et al. 2008; Luijten et al. 2011; Vara et al. 2014; Hartmann et al. 2016; Wilson et al. 2016). Using GNGT, Tamm et al. (2002) postulated an association between increasing left IFG activation and response inhibition abilities during development. Conversely, Vara et al. (2014) revealed bilateral inferior frontal activation in adolescents, but more right lateralized inferior frontal activity in adults; see Table 3 of Vara et al. (2014).

Taken together, the IFG is a crucial brain region associated with various dimensions of IC. The left IFG seems to be more related to rapid response execution (Tamm et al. 2002), the successful implementation of IC over motor responses (Swick et al. 2008) and DT (Ivancovsky et al. 2019), while the right IFG is mainly associated with unconscious, automatic, tonic inhibition and IC (Lenartowicz et al. 2011; Aron et al. 2014; Cunillera et al. 2016; Campanella et al. 2017).

Besides IFG, the right dorsolateral prefrontal cortex (rDLPFC) may be engaged in active inhibitory processing of both motor and higher level memory representations (Penolazzi et al. 2014; Friehs and Frings 2018, 2019; Sandrini et al. 2020). Using tDCS, Penolazzi et al. (2014) reported that cathodal stimulation over rDLPFC leads to decreased inhibition during the standard retrieval-practice paradigm (RPP). Also, Friehs and Frings (2018) examined the inhibitory role of rDLPFC on Stop-Signal Reaction Time (SSRT) using tDCS. They reported a reduction in SSRT and the number of omission errors after anodal tDCS (a-tDCS). The involvement of rDLPFC in monitoring the need to stop and stepping into action when top-down IC is required (Fuster 2015) is in line with the previous findings. However, a later study by Friehs and Frings (2019) did not find a modulation of error rates in any form but only a significant increase in SSRT after cathodal tDCS (c-tDCS). Sandrini et al. (2020) further verified that both the rDLPFC and right inferior parietal cortex (rIPC) represent an essential part of the fronto-basal-ganglia network, which is critical for rapid response inhibition. Accordingly, Aron et al. (2014) proposed that DLPFC implements task rules rather than inhibition. Finally, Zmigrod et al. (2015) provided direct evidence for the role of the left DLPFC (lDLPFC) in CT and DT but a mediating role of the PFC in problem solving behavior, presumably through attentional processes.

Support for the idea that IC and creativity are closely related and that both functions depend on shared neural substrates, particularly in the IFG, comes from lesion studies and studies experimentally manipulating brain regions underlying IC. For instance, lesions leading to an attenuation of cognitive inhibition allow patients to be more creative (Kapur 1996; Miller et al. 2000; Miller and Hou 2004; Seeley et al. 2008; Shamay-Tsoory 2011). Seeley et al. (2008) reported an enhancement of right hemisphere activation in a patient after progressive degeneration of the left frontal hemisphere (left IFG included). Along the same lines, Miller and colleagues reported on patients with left hemispheric degeneration (Miller et al. 1996) who developed creative abilities such as musical or artistic skills (Miller et al. 2000; Miller and Hou 2004). It might be speculated that reduced left IFG activation might have resulted in decreased IC, which in turn was associated with enhanced creativity. Accordingly, Kapur (1996) postulated a^”^paradoxical functional facilitation^”^ theory, where he explained the increment of creativity as the result of brain damage affecting areas involved in attenuation (i.e., left temporoparietal and inferior frontal regions).

Moreover, Shamay-Tsoory (2011) revealed a positive correlation between lesions in left parietal areas and increased levels of creativity. Combined, one can expect that in the process of being creative, IC might be a cognitive control mechanism important for developing original ideas or giving non-conventional answers. Therefore, reduced activation of left frontal regions (i.e., left IFG) and increased activation of right frontal regions (i.e., right IFG) should influence the creative performance. However, up to now, a causal relationship between creativity and IC has not been revealed. With this current study, we intended to establish such a causal relationship between creativity and IC, as one dimension of creative cognition (Benedek et al. 2012; Mok 2012; Cassotti et al. 2016; Khalil et al. 2019) through experimentally manipulating IC using tDCS.

Several studies used brain stimulation (i.e., tDCS) to modulate and to explore components of IC and its association with creativity (Mayseless and Shamay-Tsoory 2015; Zmigrod et al. 2015; Lucchiari et al. 2018). Findings from tDCS studies by Mayseless and Shamay-Tsoory (2015) supported a Balance Hypothesis, according to which creativity demands a balance of activation between both hemispheres of the frontal lobes (and more specifically, between the right and the left IFG). These authors applied a bilateral tDCS stimulation with the cathode over the right IFG, and the anode over the left IFG, and compared this condition with the contrary one. Results revealed increased DT scorings with left cathodal and right anodal stimulation but no effect on creativity in the reverse condition. Unimodal stimulation with either the anode or the cathode over the left or right IFG alone, however, was not sufficient to alter the creative process (Mayseless and Shamay-Tsoory 2015).

Recently, Lucchiari et al. (2018) presented a critical review of original research articles investigating the various influences of tDCS on creativity and its underlying mechanisms (cf Table 1 of Lucchiari et al. (2018)). They concluded that tDCS effects are considerably unspecific, modulating only the likelihood of more creative thinking. They further expressed the necessity for a more comprehensive framework related to creativity research and brain stimulation (Lucchiari et al. 2018).

**Table 1:** Effects of tDCS on creativity dimensions as revealed by ANCOVA analysis (cf. Fig. 3)

*F*-Statistics						
Creativity dimension	Factor	Sum Sq	*Df*	*F* value	*p* value	*p*Eta²
Fluency	Intercept	391.97	1	95.44	1.555e−11	0.731
	Baseline values*	**38.25**	**1**	**9.31 **	0.004**	**0.210**
	tDCS condition	0.51	1	0.12	0.727	0.003
	Baseline values:tDCS condition	0.22	1	0.05	0.818	0.001
	Residuals	143.74	35			
Originality	Intercept	240.56	1	39.60	3.18e−07	0.531
	Baseline values	8.75	1	1.44	0.238	0.039
	tDCS condition*	**33.25 **	**1**	**5.47 **	0.025*	**0.135**
	Baseline values:tDCS condition	0.41	1	0.07	0.797	0.002
	Residuals	212.60	35			
Flexibility	Intercept	110.58	1	135.40	1.394e-−13	0.794
	Baseline values	0.13	1	0.16	0.688	0.005
	tDCS condition	0.04	1	0.05	0.831	0.001
	Baseline values:tDCS condition	0.98	1	1.20	0.282	0.033
	Residuals	28.58	35			

* ** Statistical significance when* p* value < 0.05

To provide additional evidence for formulating such a framework, we intended to address the question of whether changes in IFG brain activity and, thus, IC would mediate changes in creativity. For that purpose, we used AUT to measure creativity in terms of DT and applied GNGT to examine the IC before and after tDCS. Tests of DT are probably one of the most commonly used assessments of creativity and do provide valuable information about creative potential. In the AUT task, participants are asked to name different, alternative uses for everyday objects. AUT measures three dimensions: fluency (i.e., number of ideas), originality (i.e., generation of uncommon ideas), and flexibility (i.e., the ability to change strategy) (Horne 1988; Chávez-Eakle et al. 2007; Scibinetti et al. 2011). From the AUT, scores for ideational fluency, ideational originality, and ideational flexibility are calculated. Ideational fluency represents the number of ideas an individual gives, while ideational originality expresses the statistical infrequency or uniqueness of ideas, and ideational flexibility depicts the number of different conceptual categories used by the individual (Runco et al. 1987; Runco and Jaeger 2012; Beketayev and Runco 2016). Thus, ideational flexibility is considered to be extremely important as it allows an individual to avoid ruts and routines when solving problems. In turn, it not only contributes to creative problem solving but is also related to adaptability and the ability to shift perspectives while solving a problem.

Based on the previous evidence, we hypothesized that, on one hand, hyperpolarization of left IFG through cathodal stimulation coupled with anodal stimulation of the right IFG (i.e., L −R +) should reveal a facilitative effect on creativity. On the other hand, creativity should be decreased in another treatment group, in which hyperpolarization of the right IFG was coupled with depolarization of the left IFG (i.e., L + R −). More precisely, we assumed that L + R − stimulation would lower AUT scores through enhancing IC as measured with the GNGT. On the contrary, the group treated with the reversed stimulation arrangement, i.e., L −R + should express higher AUT scores based on decreased IC. In particular, we expected that changes in IC induced by c-tDCS targeting the left IFG coupled with a-tDCS targeting the right IFG should result in altered originality and flexibility, but not necessarily fluency in the AUT.

## Materials and Methods

### Participants

Participants were recruited from the Jacobs University student body via emails and received course credits or monetary compensation. Treatment of the participants followed local ethical standards and German law, and followed the principles for ethical conduct on humans, as outlined in the Declaration of Helsinki (DoH), in all aspects of this study. Before the study, each individual was informed about the possibility of having different sensitivity to the tDCS stimulation. All participants underwent an eligibility screening for the tDCS procedure and signed an informed consent form. After the experiment, the participants were verbally debriefed and were informed about the results of the study via email.

Before the experiment, the participants completed a questionnaire about their mental and physical health, drug and medication use, and family history of diseases. Participants with a history of diagnosed neurological disease or psychiatric disorders, heart conditions, severe head injury, seizures (personal or in first degree relatives), recurring syncope, or learning disability were excluded from the study. Additional exclusion criteria included pregnancy, presence of metal in the face or the head (other than dental work), presence of skin conditions on the scalp or history of severe dermatitis, on-going or recent use of medical prescriptions other than contraceptives, and excessive use of alcohol on the day before the stimulation session. We excluded two participants as one had previous experience with AUT while the other got a headache due to her/his sensitivity to tDCS.

Additionally, data of one participant were not considered for analysis since the stimulation had to be terminated after 10 min due to pain.

The final sample consisted of 40 (22 male and 18 female) healthy undergraduate students between 18 and 23 years of age (average age = 19, SD = 1.48). In a randomized order, participants were assigned to one of two groups: cathodal stimulation (i.e., hyperpolarization) of left IFG combined with anodal stimulation (i.e., depolarization) of right IFG (”L −R + ”) or vice versa (i.e.,”L + R −”).

### Procedures

**Fig. 1:**
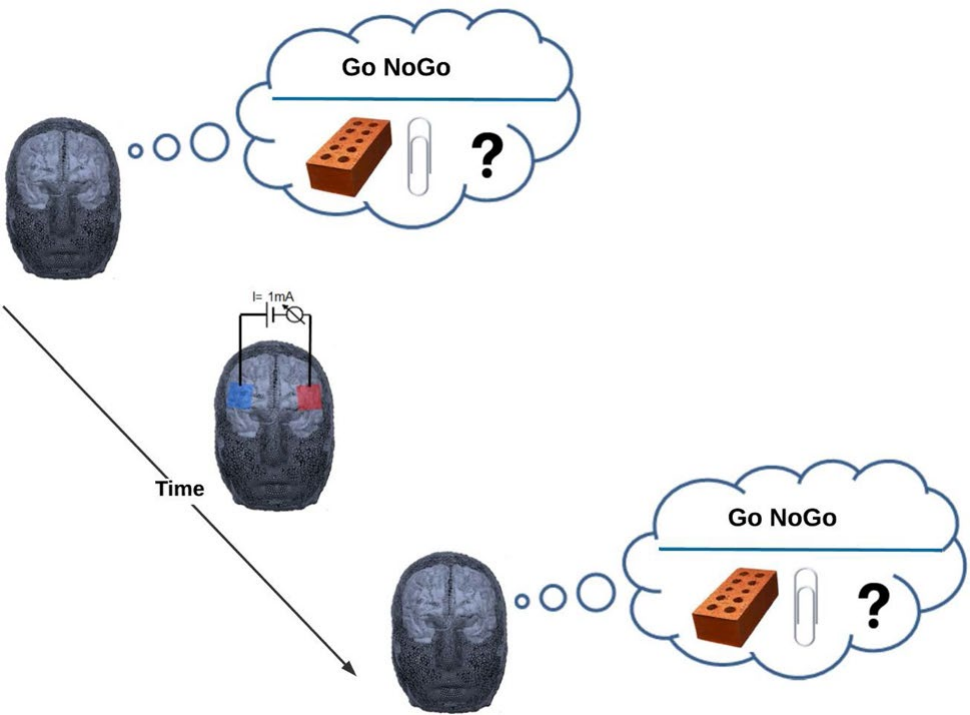
Schematic-view illustrates the experimental design. Participants’ creativity and inhibitory control (IC) were accessed by the Alternative Uses Task (AUT) and Go NoGo task (GNGT) before and after tDCS of the inferior frontal gyrus (IFG). During the AUT, participants were asked to write down creative ideas about alternative uses of two objects: A brick and a paper clip within 2 min for each object. Different words were used for the AUT in the pre- and post-test. The order of these words was counterbalanced. The task measured fluency (number of different types of categories of ideas), originality (uniqueness/novelty of the ideas), and flexibility (number of switches between different ideas). Weak direct current (1 mA) was applied between two 4 × 6 cm^2^ large wet sponge electrodes placed over F7, and F8 according to the international 10–20 EEG system for 30 min. In a randomized order, half of the participants received anodal tDCS of the right IFG and cathodal tDCS of the left IFG, whereas the other half received the opposite current flow

All subjects were naive to the tDCS procedures. The experiment took place in the exam-free period to reduce the chance of having sleep-deprived or stressed participants as it has been shown that sleep deprivation (Horne 1988; Killgore 2007) and stress (Krop et al. 1969) negatively affect DT. The participants took part in AUT and GNGT before and after brain stimulation by tDCS (cf. Fig. 1). The GNGT consistently took part after the AUT task. Potential tDCS side effects were assessed with a questionnaire administered immediately at the end of the experimental session. After the experiment, the participants were orally examined to assure that they had not been familiar with the AUT task and that they could not distinguish the stimulation conditions.

### tDCS and Electrode Placement

tDCS polarizes the brain tissue with electrical fields through two electrodes—an anode and a cathode—placed on the scalp (Cerruti and Schlaug 2009). Whereas the cathode decreases cortical excitability (hyperpolarization of cortical neurons under the cathode), thus leading to higher thresholds for the firing of action potentials (AP), the anode has an excitatory (depolarizing) effect that would cause increased firing probability of cortical neurons over time (Cerruti and Schlaug 2009; Brunoni et al. 2012; Medeiros et al. 2012). Depending on the polarity, intensity, and duration of stimulation, the effects can last for different periods—from minutes to hours (Nitsche and Paulus 2000).

A battery-driven stimulator (Schneider Electronic, Gleichen, Germany) was used for the application of tDCS over the left and right IFG. A constant current of 1 mA was applied via two saline-soaked sponge electrodes covering an area of 4 × 6 cm. tDCS was applied for 30 min with a 10 s ramp up and down. According to Nitsche and Paulus (2000) and Nitsche et al. (2003), this stimulation period should be sufficient to induce changes in cortical excitability that are stable for at least an hour.

**Fig. 2:**
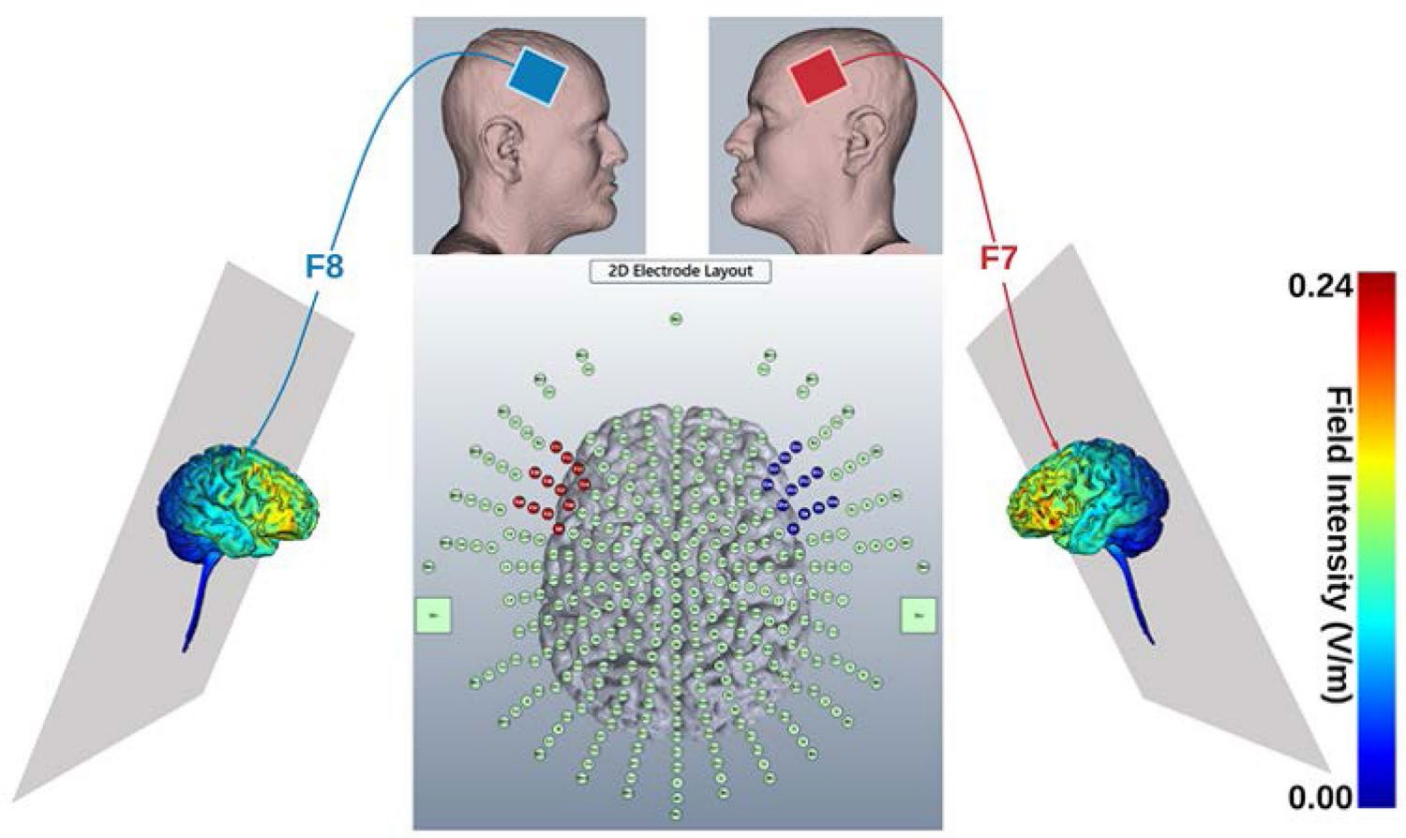
A schematic graph illustrating the modeling of current flow when applying 1.0 mA tDCS for F7 anodal (right-hand side) and P8 cathodal (left-hand-sided) stimulation. Red color points to the inward (anodal) electrical field (EF), while blue represents outward (cathodal) EF. The middle graph refers to the 2D electrode layout montage of a 338-point head model while the lateral graphs besides the head montage show EF magnitude plots. The color bar indicates the field intensity of tDCS stimulation. This model stimulation had been created using Soterix Medical High-Definition transcranial Direct Current Stimulation (HD-tDCS)

The two electrodes were placed on the scalp using the 10/20 EEG system and secured in place with electrode positioning bands. An EEG cap was used to localize and mark the positions of F7 and F8 on the scalp, which were associated with the left and right IFG, respectively (Ozawa et al. 2014; cf. Fig. 2).

We chose a bilateral bipolar-balanced montage (Neuling et al. 2012; Nasseri et al. 2015), in which we placed the electrodes symmetrically to simultaneously activate the left IFG (F7) and inhibit its right-hemispheric counterpart (F8) or the other way around. This montage is well suited to stimulate frontal brain areas without too much involvement of other cortical regions (Neuling et al. 2012). A shared prefrontal network (including left and right IFG) serves for the processes of response selection and inhibition (Goghari and MacDonald 2009). The requirement of the left IFG commonly occurs when controlled responses were required, probably due to its role in the response selection process. Nevertheless, the involvement of the right IFG occurs more when task demands are more potent for response inhibition. Furthermore, the current strength in both hemispheres (the left IFG and the right IFG) is rather similar, which makes such a bilateral configuration especially suitable to test the Balance Hypothesis (Mayseless and Shamay-Tsoory 2015). Finally, a similar bilateral configuration was used previously in several studies on creativity (Chrysikou et al. 2013; Mayseless and Shamay-Tsoory 2015; Lucchiari et al. 2018).

## Behavioral Tasks

### Alternative Uses Task (AUT)

During the AUT task, participants were asked to write down creative ideas about alternative uses of two objects (brick and paperclip) within 2 min (cf., Mayseless and Shamay-Tsoory 2015). Before executing the task, an example of the uses for a newspaper was displayed to familiarize them with the task. Different words were used for the AUT in the pre- and post-test. The order of these words was counterbalanced. The task measured fluency (number of different types of ideas' categories), the novelty of the ideas (uniqueness/originality) (Carroll and Guilford 1968), and flexibility (the ability to change strategy) (Horne 1988).

We used the standard AUT scoring method from the Runco Creativity Assessment Battery (Runco and Jaeger 2012; Beketayev and Runco 2016). We computed the fluency score as the number of answers given; hence, it measures the ideational productivity, only. Comparatively, the originality score was computed from the statistical infrequency of an answer within the pool of answers. If an idea was unique, it got 100 pts. If an idea was given three times, it got 100/3 = 33.3 pts. If an idea was given a hundred times, it got 100/100 = 1 pt. Lastly, each idea was assigned to an a priori conceptual category (one set of categories for each task), and the flexibility score was calculated as the number of categories used by the participant. This method has demonstrated good inter-rater and inter-item reliability in numerous investigations (cf., Beketayev and Runco 2016).

### GO NOGO Task (GNGT)

The GNGT was performed as described in the experiment by Swick et al. (2008). Lower case black letters, font Times New Roman, size 140, were presented on a white screen. The “No-Go” stimulus was the letter “x” while all the other letters of the alphabet were “Go” stimuli. The participants were instructed to press a lever with the index finger as soon as they would see a Go stimulus and inhibit their response when presented with a No-Go stimulus. The duration of stimuli presented at the center of the computer screen was 200 ms, and interstimulus intervals were 1500 ms. There were two types of paradigms in which the proportion of Go to NoGo trials was either 50/50 or 90/10. In the 50/50 paradigm, out of the 140 trials in a block, 70 trials were a “Go” and 70 trials were a NoGo stimulus. In the 90/10 paradigm, 14 trials were NoGo stimulus, and 126 trials were “Go” trials. The participants were familiarized with the task by a short practice set of 30 trials (15 Go and 15 No-Go stimuli, randomly intermixed) and had a short break between the 2 blocks.

On the principle of signal detection theory (SDT; Green and Swets 1966), we used hit rate (HR) and false alarm (FA) rate to calculate the sensitivity index *d*-prime (*d*′) for accuracy*,* where increasing values of *d*′ refer to higher sensitivity to a given signal (i.e., GO stimuli). Both HR and FA were adjusted to avoid extreme values of 0 and 1, using the linear log approach of Hautus (1995), whereby 0.5 is added to the number of hits, and the number of false alarms and 1 added to the number of Go and NoGo trials. *d*’ was computed as the difference between the standardized (Z-transformed) probability of the HR and FA rate: (*d*′ = *z*HR – *z*FA; Stanislaw and Todorov 1999). Reaction times (RTs) were calculated for correct trials.

### Data Analysis and Statistics

We performed all statistical analyses using Graph Prism (GraphPad Software, San Diego, California USA, www.graphpad.com) and R version 3.5.1 (https://www.R-project.org/) software packages. Results were considered statistically significant with a *p* value < 0.05. To test the hypothesized mediation effect of tDCS-induced changes in IC on creativity, we followed a three-step procedure.

- In the first step, we aimed at establishing an effect of tDCS on DT, as revealed by the AUT. ANCOVAs were performed separately for the creativity scores fluency, originality, and flexibility with tDCS condition ( L−R + , L + R −) as between subjects factor, post-tDCS creativity scores as dependent variables, and baseline values as covariates. This procedure better controls baseline differences in intervention studies than repeated measures ANOVA (Twisk et al. 2018). Furthermore, planned comparisons were performed between pre- and post-values separately for the two tDCS conditions using paired-samples t tests.
- In a second step, we similarly performed separate ANCOVAs for RT and *d*′ as obtained in GNGT with post-tDCS values as dependent variables, pre-tDCS values as covariates, and tDCS (L −R + , L + R −) and GNGT condition (50/50, 90/10) as between and within-subject factors, respectively. Again, planned comparisons were performed between pre- and post-values using paired-samples *t* tests.
- In the final step, pre-to-post-tDCS changes in GNGT (*z*-transformed, centered) were introduced as covariates in the first model. A mediation effect of tDCS-induced IC changes on creativity would be revealed by attenuating a potential direct effect of tDCS on creativity scores, as found in the first analysis step (a). This third ANCOVA model would allow for testing moderation effects of IC changes on changes in creativity induced by tDCS. Such moderation effects would be identified as interaction effects of tDCS and pre-to-post changes in GNGT on creativity scores and can be interpreted as that changes in creativity depend on the strength or direction of changes in GNGT without a direct causal relationship (i.e., increase or decrease in GNGT is associated with an increase or decrease in creativity).

## Results

### Creativity (AUT)

**Fig. 3:**
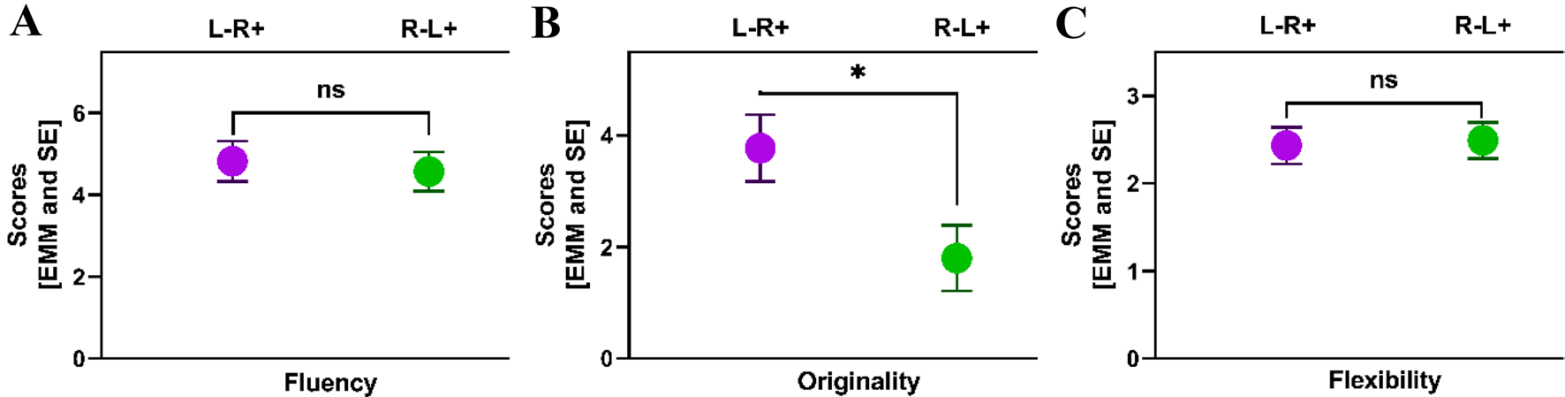
Creativity scores (*y* axes) achieved by the participants during the AUT task after tDCS of left and right IFG. L − R + relates to anodal stimulation of the right inferior frontal gyrus (IFG) (coupled with cathodal stimulation of the left IFG) while L + R − refers to anodal stimulation of the left IFG (coupled with cathodal stimulation of the right IFG). Panels (**a**–**c**) refer to the scores for fluency, originality, and flexibility, respectively. Shown are estimated marginal means and SE at a fixed level of the respective pre-value as a covariate in the ANCOVA. There was a significant effect of tDCS condition on originality, only. Cf., Table 1, for detailed statistics)

Results for the creativity tests are illustrated in Fig. 3a–c. For originality, but not fluency or flexibility, scores (estimated marginal means adjusted for baseline values) were lower after L + R − than L −R + . One-way repeated measures ANCOVA revealed a significant effect of tDCS condition on originality (*F*(1,35) = 5.4746, *p* = 0.025, *p*Eta^2^ = 0.135) but no main or interaction effects of the covariate (cf. Table 1 for detailed statistics). While for flexibility, no significant effect was revealed, for fluency, there was only an effect of the covariate (i.e., baseline values; *F* (1,35) = 9.3138, *p* = 0.004, *p*Eta^2^ = 0.210). Planned comparisons for L + R − confirmed a considerable trend toward a significant tDCS session effect for flexibility (*t* = 1.917, *df* = 40, *p* = 0.062) but not for originality or fluency (cf., supplementary Fig. 1).

### Inhibitory Control (IC)

**Fig. 4:**
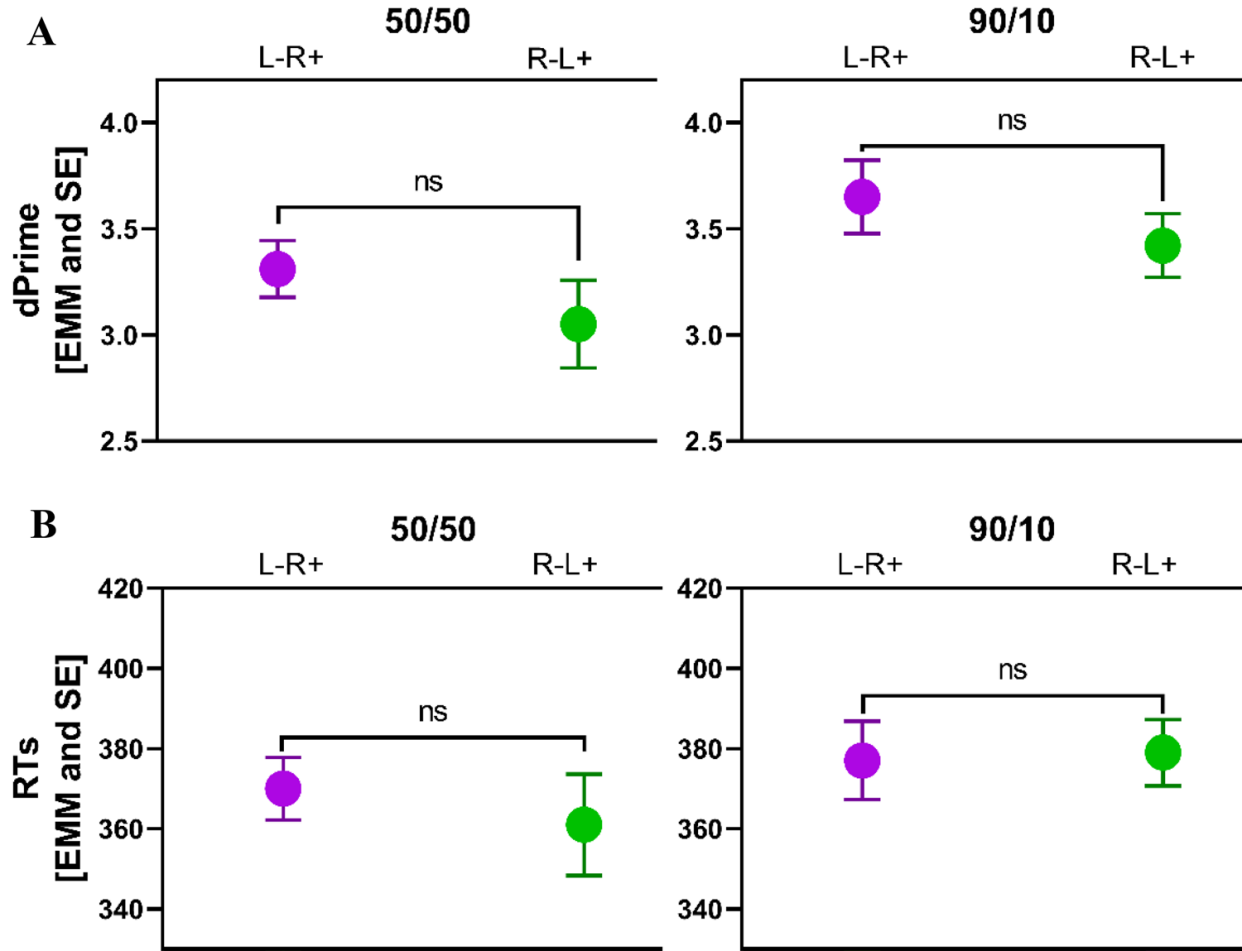
**a**, **b** show dprime values and reaction times (RTs), respectively, for the two conditions of the GO NOGO task (i.e., 50:50 and 90:10) pre and post tDCS. L − R + relates to anodal stimulation of the right inferior frontal gyrus (IFG) (coupled with cathodal stimulation of the left IFG) while L + R- refers to anodal stimulation of the left IFG (coupled with cathodal stimulation of the right IFG). Shown are estimated marginal means and SE at a fixed level of the respective pre-value as a covariate in the ANCOVA. Cf., Table 2 for detailed statistics

Results for the GNGT are illustrated in Fig. 4a, b. While *d′* values seem to be lower aftr − L + R − as compared to L −R + in both conditions, for RT, this is true only for the 50/50 condition. ANCOVA, however, did not confirm any significant main or interaction effect of tDCS on GNGT performance, neither any other significant effect (cf. Table 2 for detailed statistics). Planned comparisons between pre- and post-tDCS measurements as well did not reveal any session effects (cf. supplementary Fig. 2).

**Table 2:** Effects of tDCS on GNGT as revealed by ANCOVA analysis (cf. Fig. 4)

*F*-Statistics						
GNGT	Factors	Sum Sq	*Df*	*F* value	*p* value	*p*Eta²
RT	Intercept	2123	1	1.73	0.192	0.024
	Baseline values*	20633	1	16.84	0.000***	0.194
	tDCS condition	1186	1	0.97	0.329	0.013
	GNGT condition	104	1	0.08	0.772	0.001
	Baseline values:tDCS condition	1250	1	1.02	0.316	0.014
	Baseline values:GNGT condition	13	1	0.01	0.917	0.000
	tDCS condition:GNGT condition	44	1	0.04	0.849	0.000
	Baseline values:tDCS condition:GNGT condition	95	1	0.08	0.782	0.001
	Residuals	85788	70			
dPrime	Intercept	0.38	1	1.18	0.281	0.016
	Baseline values*	3.09	1	9.58	0.003**	0.120
	tDCS condition	0.07	1	0.22	0.643	0.003
	GNGT condition	0.02	1	0.07	0.794	0.001
	Baseline values:tDCS condition	0.01	1	0.02	0.895	0.000
	Baseline values:GNGT condition	0.00	1	0.01	0.930	0.000
	tDCS condition:GNGT condition	0.14	1	0.43	0.512	0.006
	Baseline values:tDCS condition:GNGT condition	0.16	1	0.48	0.489	0.007
	Residuals	22.59	70			

** *** Statistical significance when* p* value < 0.05

### Interaction between Creativity (AUT) and Inhibitory Control (GNGT)

**Fig. 5:**
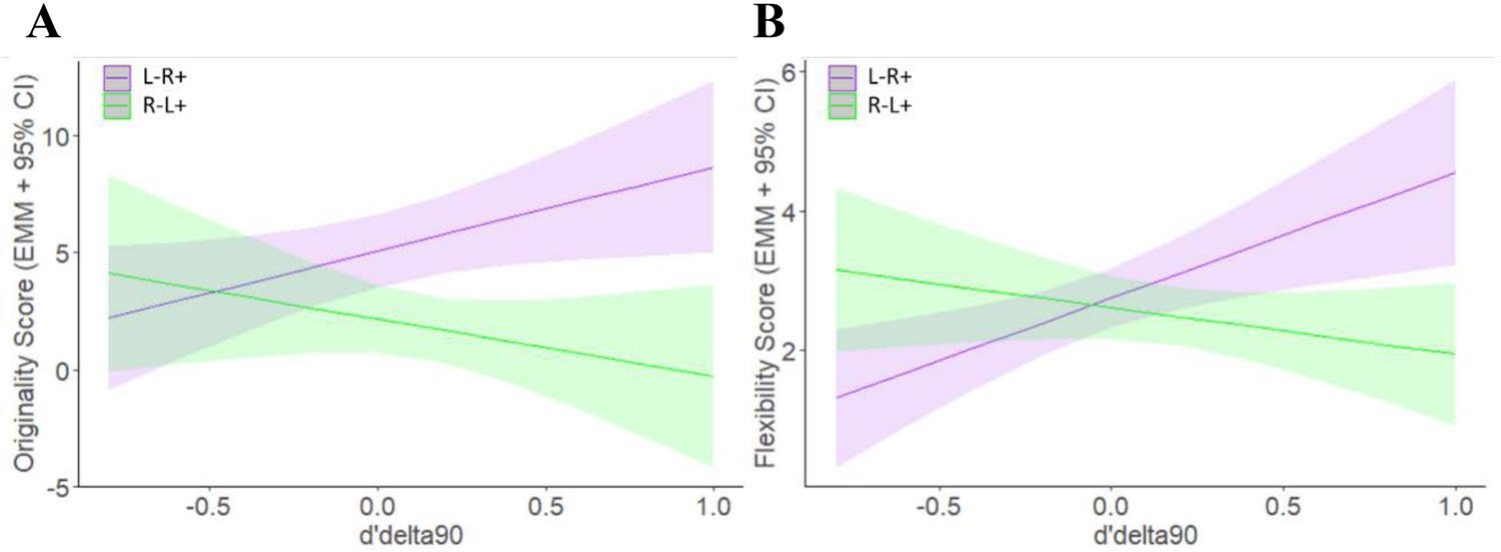
Association between changes in dPrime (*d*’delta90; 90/10 condition of GNGT) and originality (**a**) and flexibility (**b**) scores (estimated marginal means) after tDCS. Linear regression lines with 95% confidence intervals are displayed. L − R + relates to anodal stimulation of the right inferior frontal gyrus (IFG) (coupled with cathodal stimulation of the left IFG) while L + R − refers to anodal stimulation of the left IFG (coupled with cathodal stimulation of the right IFG). ANCOVA expressed a significant interaction effect of d’delta90 and type of tDCS on post tDCS creativity scores for originality (*F* (1,19) = 5.521, *p* = 0.030, *p*Eta^2^ = 0.225) and flexibility (*F* (1,19) = 9.901, *p* = 0.005, *p*Eta^2^ = 0.343): at positive levels of *d*’delta90 (increased GNGT performance after tDCS), creativity scores were higher after L − R + stimulation than after L + R − stimulation. No difference between stimulation conditions was revealed when there was no change or a decline in GNGT (negative *d* ‘delta90 values; cf. Table 3 for detailed statistics)

ANCOVA with post-tDCS creativity scores as dependent variable, tDCS condition as between subjects factor, and pre-tDCS scores and changes in IC (RTdelta50, RTdelta90, d’delta50, d’delta90) as covariates revealed a significant interaction effect of d’delta90 and type of tDCS on post-tDCS creativity scores for originality (*F*(1,19) = 5.521, *p* = 0.030, pEta^2^ = 0.225) and flexibility (*F*(1,19) = 9.901, *p* = 0.005, *p*Eta^2^ = 0.343; for detailed statistics, cf. Table 3): as illustrated in Fig. 5, at positive levels of d’delta90 (increased GNGT performance after tDCS), creativity scores were higher after L −R + stimulation than after L + R − stimulation. No difference between stimulation conditions was revealed when there was no change or a decline in GNGT.

**Table 3:** Interaction between creativity dimensions and GNGT as revealed by ANCOVA analysis (cf. Fig. 5)

*F*-Statistics						
Creativity dimension	Factors	Sum Sq	*Df*	*F* value	*p* value	*p*Eta²
Fluency	Intercept	0.09	1	0.02	0.897	0.001
	Baseline values*	37.06	1	7.38	0.014*	0.280
	tDCS condition	0.69	1	0.14	0.715	0.007
	d'delta 50	0.63	1	0.13	0.728	0.006
	d'delta90	0.35	1	0.07	0.794	0.004
	d’RT50	0.06	1	0.01	0.918	0.000
	d’RT90	0.03	1	0.01	0.936	0.000
	Baseline values:tDCS condition	0.25	1	0.05	0.825	0.003
	Baseline values: d'delta 50	6.92	1	1.38	0.255	0.067
	Baseline values: d'delta 90	0.61	1	0.12	0.732	0.006
	Baseline values: d’RT50	7.89	1	1.57	0.225	0.076
	Baseline values: d’RT90	0.30	1	0.06	0.810	0.003
	tDCS condition:d'delta 50	0.13	1	0.03	.873	0.001
	tDCS condition: d'delta 90	2.16	1	0.43	0.519	0.022
	tDCS condition: d’RT50	0.00	1	0.00	0.980	0.000
	tDCS condition: d’RT90	4.24	1	0.84	0.370	0.042
	Baseline values: tDCS condition: d'delta 50	9.16	1	1.82	0.193	0.087
	Baseline values: tDCS condition: d'delta 90	0.01	1	0.00	0.960	0.000
	Baseline values: tDCS condition: d’RT50	5.40	1	1.07	0.313	0.053
	Baseline values: tDCS condition: d’RT90	0.02	1	0.00	0.954	0.000
	Residuals	95.45	19			
Originality	Intercept	14.30	1	2.54	0.127	0.118
	Baseline values	1.34	1	0.24	0.631	0.012
	tDCS condition*	45.80	1	8.13	.010*	0.300
	d'delta 50	8.16	1	1.45	0.243	0.071
	d'delta90	1.14	1	0.20	0.657	0.010
	d'RT50	1.07	1	0.19	0.668	0.009
	d'RT90	1.58	1	0.28	0.602	0.015
	Baseline values:tDCS condition	14.58	1	2.59	0.124	0.119
	Baseline values: d'delta 50	9.97	1	1.77	0.199	0.085
	Baseline values: d'delta 90	4.79	1	0.85	0.368	0.043
	Baseline values: d'RT50	8.97	1	1.59	0.222	0.077
	Baseline values: d'RT90	8.72	1	1.55	0.228	0.075
	tDCS condition:d'delta 50	16.04	1	2.85	0.108	0.130
	tDCS condition:d'delta 90*	31.08	1	5.52	0.030*	0.225
	tDCS condition: d’RT50	7.93	1	1.41	0.250	0.069
	tDCS condition: d'RT90	11.61	1	2.06	0.167	0.098
	Baseline values: tDCS condition: d'delta 50	9.66	1	1.72	0.206	0.083
	Baseline values: tDCS condition: d'delta 90	0.10	1	0.02	0.896	0.001
	Baseline values: tDCS condition: d'RT50	1.07	1	0.19	0.668	0.009
	Baseline values: tDCS condition: d'RT90	1.57	1	0.28	0.603	0.014
	Residuals	106.96	19			
Flexibility	Intercept	1.64	1	2.85	0.108	0.130
	Baseline values	1.56	1	2.70	0.116	0.125
	tDCS condition	0.13	1	0.22	0.646	0.011
	d'delta 50	2.14	1	3.70	0.070	0.163
	d'delta90	1.19	1	2.06	0.167	0.098
	d'RT50	0.39	1	0.68	0.420	0.034
	d'RT90	0.32	1	0.56	0.463	0.029
	Baseline values: tDCS condition	0.86	1	1.49	0.237	0.073
	Baseline values: d'delta 50	0.01	1	0.02	0.879	0.001
	Baseline values: d'delta 90	0.07	1	0.12	0.735	0.006
	Baseline values: d'RT50	0.32	1	0.56	0.464	0.028
	Baseline values: d'RT90	0.00	1	0.00	0.952	0.000
	tDCS condition: d'delta 50	1.51	1	2.61	0.122	0.121
	tDCS condition: d'delta 90*	5.71	1	9.90	0.005**	0.343
	tDCS condition: d’RT50	1.29	1	2.24	0.151	0.106
	tDCS condition: d'RT90	0.59	1	1.02	0.326	0.051
	Baseline values: tDCS condition: d'delta 50	0.06	1	0.11	0.748	0.006
	Baseline values: tDCS condition: d'delta 90*	4.76	1	8.25	0.009**	0.303
	Baseline values: tDCS condition: d'RT50	0.01	1	0.02	0.879	0.001
	Baseline values: tDCS condition: d'RT90	1.77	1	3.07	0.096	0.139
	Residuals	10.97	19			

* ** Statistical significance when* p* value < 0.05

For flexibility, there was further a three-fold interaction effect of tDCS condition, *d*’delta90, and pre-tDCS score (*F*(1,19) = 8.251, *p* = 0.010, *p*Eta^2^ = 0.303) as well as marginally significant effects of *d*’delta50 (*F*(1,19) = 3.6986, *p* = 0.070, *p*Eta^2^ = 0.163) and the interaction between tDCS condition, RTdelta90, and baseline values (*F*(1,19) = 3.0695, *p* = 0.096, *p*Eta^2^ = 0.139). Considering originality, the main effect of tDCS condition, as revealed by analysis (a) above, was confirmed (*F*(1,19) = 8.135, *p* = 0.010, *p*Eta^2^ = 0.300). Regarding fluency, only baseline values revealed a significant effect on post-tDCS scores (F (1,19) = 7.378, *p* = 0.014, *p*Eta^2^ = 0.280).

Post hoc analysis (separate ANCOVA for L + R − and L −R + with pre-scores and *d*’delta 90 as covariates) revealed a positive effect of *d*’delta90 on flexibility scores after L−R + (F (1,19) = 4.781, *p* = 0.042, *p*Eta^2^ = 0.210), but not after L + R−. For originality, a marginally significant negative effect of d’delta90 on originality was revealed for L + R− (*F*(1,19) = 3.590, *p* = 0.075, *p*Eta^2^ = 0.174), but not for L−R + .

## Discussion

Our study had two primary goals: first, to investigate the effect of tDCS on divergent thinking (DT; through AUT) and inhibitory control (IC; through GNGT), and second, to explore the relationship between AUT, GNGT, and the activity of the left and right IFG.

Concerning the first goal, in line with other studies (Mayseless and Shamay-Tsoory 2015; Lucchiari et al. 2018; Ivancovsky et al. 2019) and, in an agreement with the Balance Hypothesis (Mayseless and Shamay-Tsoory 2015), we found an enhancement of ideational originality after L −R + (facilitation of right IFG and inhibition of left IFG) but not after the opposite stimulation regime. However, the study by Mayseless and Shamay-Tsoory (2015) showed only a trend in increasing performance in all three dimensions of AUT, i.e., ideational fluency, originality, and flexibility, while we did not find any direct effect of tDCS on flexibility and fluency.

Regarding IC, no direct effect of tDCS could be revealed. This result might be due to the simplicity of GNGT, i.e., the ease to discriminate between Go and NoGo stimuli (Sallard et al. 2018). Moreover, the lack of offline effects of tDCS on GNGT performance reported by Sallard et al. (2018) corroborates findings by previous tDCS/GNGT studies stimulating the right IFG (Cunillera et al. 2016; Campanella et al. 2017). Research has demonstrated that offline stimulation improved IC during a SST (Cai et al. 2016) but not GNGT (Campanella et al. 2017). Similarly, using SST, other studies found an enhancement of inhibition accuracy after tDCS over the right IFG (Jacobson et al. 2011; Ditye et al. 2012; Stramaccia et al. 2015; Cai et al. 2016). While Cunillera et al. (2014) reported a simultaneous modulation of two kinds of inhibition processes (reactive and proactive inhibition processes), by a-tDCS on the right IFG, using SST and GNGT, respectively. Additionally, Stramaccia et al. (2017) revealed that tDCS over the right IFG disrupts control over interference using memory inhibition tasks. Therefore, one should take into consideration the type of task used to measure IC.

Other factors that might explain our results regarding the effect of tDCS on GNGT could be baseline activity levels. A study by Sallard et al. (2018) suggested that the baseline level of engagement of the brain areas of interest might be a critical factor in determining the functional effect of tDCS, which was confirmed by changes in the BOLD signal after a-tDCS manifesting only under conditions of low task-related activity.

With regards to the second goal, regarding the relationship between AUT, GNGT, and the activity of the left and right IFG, as we did not find a direct effect of tDCS on GNGT, no mediation effect of IC can be assumed. However, ANCOVA analysis showed a potential moderation effect (as indicated by a significant interaction effect of tDCS condition and change in *d’*): for both, originality and flexibility, but not fluency, post-stimulation scores were higher after L −R + as compared to L + R − conditions, only when associated with increased IC as revealed by higher *d’* (90/10 condition). It is not surprising that this moderation effect was found only for the 90/10 but not the 50/50 condition of the GNGT, as the demand on IC resources is higher in the 90/10 condition (Swick et al. 2008). No difference between stimulation conditions was revealed when there was a decline in GNGT (negative *d*’delta90 values; cf. Fig. 5).

A potential explanation for the described moderation effect, i.e., that tDCS is only effective in enhancing originality and flexibility through left cathodal and right anodal tDCS when *d’* in the GNGT is on an enhanced level. This enhanced *d’* level might reflect a specific mind state or another latent factor that facilitates or attenuates the effects of tDCS on creativity (i.e., the moderation effect of *d’*). Dependencies of the tDCS effect on the subjects' neurocognitive states have been suggested previously (Learmonth et al. 2015; Hsu et al. 2016). We admit that these arguments are speculative and need to be further investigated. Consequently, better control of all the previous aspects could thus help to improve the reliability of the effects of tDCS on brain activity, and by extension, on its behavioral consequences.

In conclusion, this study emphasizes the effectiveness of shifting activity from left to right IFG through tDCS for creative performance in a DT task. Of interest, tDCS stimulation did not significantly modulate performance in all three AUT dimensions. c-tDCS targeting the left IFG associated with a-tDCS over the right IFG resulted in increasing originality and flexibility, but not fluency in the AUT.

### Open Question, Limitations and Future Directions

Our current study highlighted the possibility of a latent factor (LF) that determines the moderation effect of *d*′ induced by tDCS on creativity (i.e., either facilitation or attenuation). Therefore, it would be of great interest to explore such factor (s) in future studies. Such latent factors could relate to the biophysical properties of the tissue (and thus the efficiency of the tDCS) or individual differences in cognitive status or mindset.

Regarding the tDCS protocol, we relied on using offline tDCS. Thus, it would be interesting to examine whether offline vs. online tDCS would result in a similar effect on AUT and GNGT and their associated functional activity. Also, due to this specific electrode montage (i.e., bilateral bipolar-balanced montage) that we applied in our study, and the non-focal nature of tDCS, we cannot rule out that observed results might reflect a combined effect of stimulation of the IFG and other regions of the frontal cortex, such as the fronto-polar region. Thus, it would be fascinating to elaborate on the tDCS effects on brain activity and network connectivity underlying these performances in the AUT and the GNGT. Lastly, we could not ignore the impact of potential individual differences and variations in the mind states on manipulating creativity through tDCS. We used the sample size of *N* = 40, which is relatively similar to what had been used in several previous publications related to original creativity research conducted using tDCS techniques (see Table 1 of Lucchiari et al. 2018). However, this sample size might be at the lower limit of our current observation.

## Electronic supplementary material

Below is the link to the electronic supplementary material.

Supplementary file1 (DOCX 210 kb)

## Abbreviations

AP: Action potentials
AUT: Alternative uses task
DT: Divergent thinking
EFs: Executive functions
GNGT: Go No Go task
IC: Inhibitory control
IFG: Inferior frontal gyrus
PFC: Prefrontal cortex
rDLPFC: Right dorsolateral prefrontal cortex
tDCS: Transcranial direct current stimulation
