# Supplementary material for: Effects of transcranial direct current stimulation of left and right inferior frontal gyrus on creative divergent thinking are moderated by changes in inhibition control
Source: Brain Struct Funct. 2020 Jun 17;225(6):1691–704. doi: 10.1007/s00429-020-02081-y (PMC7321900; doi:10.1007/s00429-020-02081-y)
Supplement: Supplementary file 1 — Supplementary file1 (DOCX 210 kb) [file 429_2020_2081_MOESM1_ESM.docx]

Supplementary Fig.1
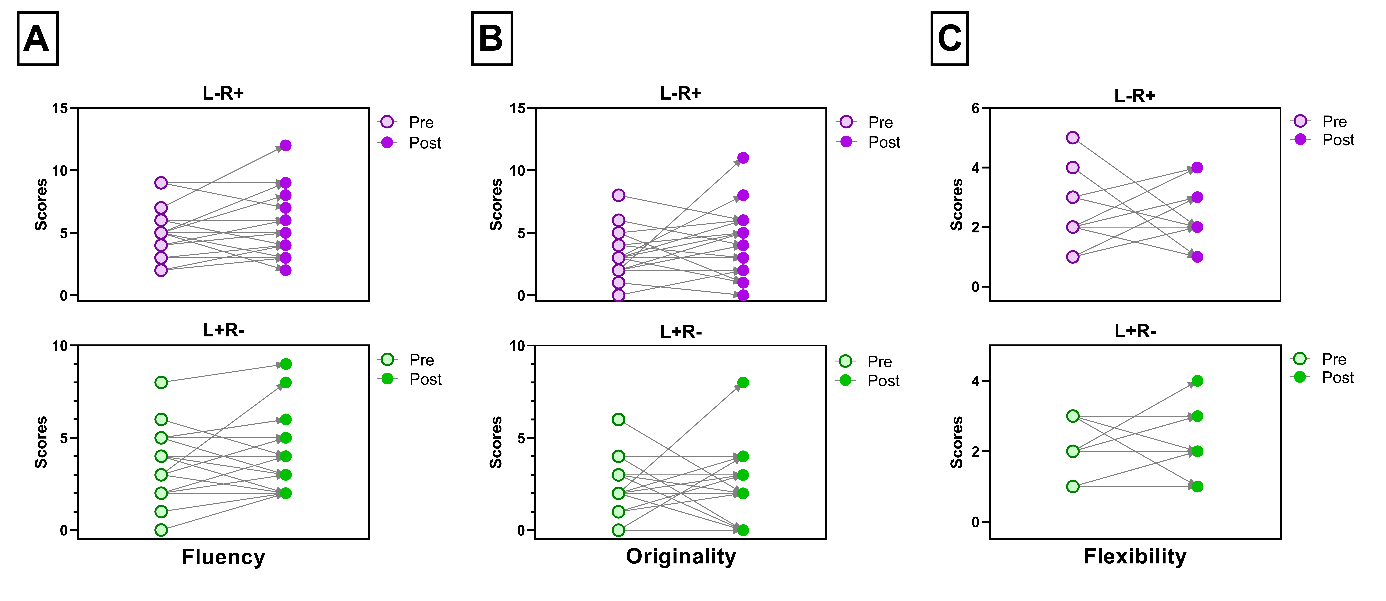


Supplementary Fig.1: Panels (A, B, and C) illustrate the pre to post tDCS changes for fluency, originality, and flexibility, respectively, and for the two tDCS conditions L-R+ (purple) and L+R- (green). (cf. Table 1, for detailed statistics.)

Supplementary Fig.2
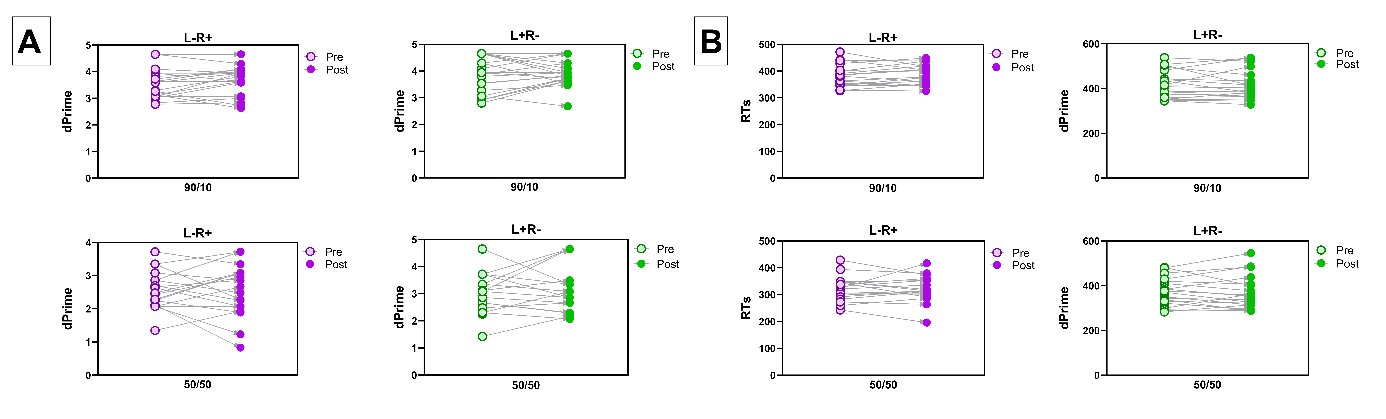


Supplementary Fig.2: Panels (A and B) illustrate the pre to post tDCS changes for dPrime and reaction times, respectively (upper part: 90/10 condition, lower part: 50/50 condition), and for the two tDCS conditions L-R+ (purple) and L+R- (green). (cf., Table 2, for detailed statistics).
